# Key-in-session identity negotiations in a first line treatment for adult anorexia nervosa

**DOI:** 10.1186/s40337-024-00979-0

**Published:** 2024-01-31

**Authors:** Lauren Heywood, Janet Conti, Stephen Touyz, Sloan Madden, Phillipa Hay

**Affiliations:** 1 School of Psychology, Western Sydney University, Sydney, Australia; 2 Translational Health Research Institute, Western Sydney University, Sydney, Australia; 3 School of Medicine, Western Sydney University, Sydney, Australia; 4 Inside Out Institute, University of Sydney and Sydney Local Health District, Sydney, Australia; 5 School of Medicine and Heath, University of Sydney, Ramsay Clinic Northside, Sydney, Australia

**Keywords:** Eating disorders, Anorexia nervosa (AN), CBT-AN, Identity negotiations, Compulsive exercise activity therapy, Narrative therapy

## Abstract

**Background:**

Exploration of client identity negotiations during treatment for Anorexia Nervosa (AN) is a relatively new area of research. Research suggests that difficulties with identity negotiations may present as a barrier to treatment. This study sought to explore individuals’ identity negotiations during therapy sessions using Compulsive Exercise Activity Therapy (LEAP) combined with Cognitive Behaviour Therapy for Anorexia Nervosa (CBT-AN). Analysis focused on moments in therapy where individuals’ identities were dominated or defined by AN and where alternative identities could be generated.

**Method:**

40 in-session transcripts from sessions at early, mid and end points of the CBT-AN (with LEAP) treatment were qualitatively analysed for nine of the 78 participants in the original randomised control trial. Through a constructivist framework, thematic analysis was used to identify surface and latent meanings and discursive material participants used to negotiate their identities in the context of therapy sessions.

**Results:**

Analysis of in-therapy transcripts generated two themes pertaining to identity negotiations: (1) troubled identities and (2) rebuilding identities and lives outside of AN. Early therapy sessions explored fragmented and AN dominated identities, including how AN was troubling to participants’ sense of self, contributed to conflicted identities, positioned them outside of normality, and was associated with isolated and othering identities. Within therapy sessions, participants engaged in a recursive process of shifting relationships with AN and themselves and building identities and lives outside of the AN identity. This included generating hopes for recovery and the future more frequently in mid- to late- therapy sessions.

**Conclusion:**

Identity negotiations evident in the therapeutic conversations aligned with the key components of the CBT-AN intervention, including addressing (1) the characterisation of oneself as ‘an anorexic’ and (2) the diversification of roles and activities to broaden and enhance self-concepts. Future developments of therapeutic interventions for AN would benefit from greater consideration of ways to assist individuals to more comprehensively address problematic identities, including uncovering identities hidden by the AN identity and generating preferred identities.

*Trial Registration*: Ethics approval was obtained at the time of the initial study and for this embedded research by the HREC at the Western Sydney University (HR777332).

**Supplementary Information:**

The online version contains supplementary material available at 10.1186/s40337-024-00979-0.

## Abstract (plain language summary)

Current psychological therapies for Anorexia Nervosa (AN) have recently identified that the sense of AN as part of a person’s identity, or who they understand themselves to be, may pose barriers for treatment. In this study, therapy session transcripts from previous research using Compulsive Exercise Activity Therapy (LEAP) combined with Cognitive Behaviour Therapy for AN (CBT-AN) were thematically analysed to explore participants’ experiences of identity shifts in-session. Particular attention was paid to moments in therapy where individuals’ identities were heavily influenced by AN and moments where alternative and preferred identities were generated. Outcomes from this study suggested that the process of participants negotiating their identities outside of AN was a gradual and repeated one throughout treatment, which involved building hopes for recovery and a future less dominated by AN. Findings from this research support the need for future treatments to broaden their scope to more comprehensively explore changes in identity during intervention, particularly in building new identities outside of the AN identity.

**Supplementary Information:**

The online version contains supplementary material available at 10.1186/s40337-024-00979-0.

## Background

Anorexia Nervosa (AN) is characterised by persistent restriction of energy intake relative to needs leading to a significantly low weight, with fear of weight gain and a disturbance in self-perception of actual weight or body shape [1]. With a lifetime prevalence rate of 1.4% in females and 0.2% in males [2], AN is the third most chronic illness among females and amongst the most fatal of psychiatric illnesses [3].

### Compulsive exercise behaviour

Up to 81% of people with AN engage in excessive or compulsive exercise [4, 5], and this is often one of the last symptoms to subside during treatment [6]. Compulsive exercise behaviour has been traditionally understood as difficult to treat and has been linked to an increased risk of relapse, poorer outcomes for recovery, and can be symptomatic of a longer-term more chronic experience of AN [7, 8]. Previous attempts to reduce compulsive exercise within AN treatment have conceptualised excessive exercise as a compensatory behaviour to account for calories consumed [9]. Recent research has indicated that exercise has a more active role in the conceptualisation and maintenance of AN, specifically in that it regulates problematic mood states and allows a person to potentially avoid or escape negative experiences or interpersonal conflict [8, 10]. Current cognitive-behavioural models [7, 11] of compulsive exercise assert that individuals who engage in excessive exercise are often found to have high standards and increased self-criticism, in addition to pervasive negative self-concepts seen as intrinsic to their identity. As such, engagement in compulsive exercise may function as a means of negotiating meanings for a person’s sense of self and identity, where low self-esteem and negative identities are addressed through a focus on physical appearance or competency, which are further reinforced by recognition and praise from family or friends. Likewise, compulsive exercise may be related to a person’s social and relational world, allowing them to engage in physical activities with others, or to feel satisfaction when high standards are achieved [12].

The role that compulsive exercise plays in individuals’ sense of self and identity is thought to contribute to the egosyntonic nature of AN more broadly [13]. AN, including compulsive exercise, may provide a variety of functions that are perceived as protective to the individual, such as a sense of control and structure, feelings of achievement and communication of intense feelings to others [13]. This egosyntonicity [14] and identity investment in the AN experience [15–18] speaks to the need to understand the role of identity in the AN experience and to address this in treatment [14]. Despite this, treatment for individuals with AN who engage in compulsive exercise has also received little previous clinical or research attention, even with indication that exercise may have a more active role in the conceptualisation of AN, including identity negotiations [8, 19]. Meyer et al. [8] have developed a novel therapy that seeks to fill this gap by treating compulsive exercise in populations of AN titled the Compulsive Exercise Activity Therapy (LEAP). LEAP seeks to address the role of compulsive exercise as a maintaining factor for AN and has been integrated with an established manualised CBT program for AN [20].

### Treatment for AN

Many individuals with AN do not engage in specialised treatment and dropout rates for those who do are high [21]. Current treatments for AN are provided in a range of inpatient and outpatient settings that employ a combination of CBT and family-based therapy (FBT) approaches, particularly for adolescents [22, 23]. Whilst best practice specialist treatments for AN show significant improvement over treatment as usual conditions [21, 24, 25], effectiveness is limited with recovery rates varying from between 30 and 60% [21]. Likewise, CBT for adult AN as an outpatient intervention has been described as having limited clinical efficacy [23] and other research has suggested that there is currently no evidence-based treatment for adult AN [26]. Although individuals may gain weight during inpatient programs, relapse rates are high following discharge and such programs may fail to address the underlying psychological aspects of the disorder [14, 23, 26].

### Identity and recovery in AN

Research studies into treatments for AN have focused predominantly on the effectiveness of therapy for AN from a medical perspective, such as weight gain and decrease in ED symptomatology [24, 27, 28]. Medical discourse also constructs the AN identity as a ‘disordered identity’ [15] with treatments tending to focus on the illness and amelioration of symptoms. Parallel to this, there has been increased interest in research that explores the meaning of AN to the experiencing person. This qualitative research has been summarised in metasyntheses, including one analysis that concluded that AN is a “complex phenomenon in which the question of identity emerges, along with its connection to outcome” [16] (p. 46). In other words, an identity (or who a person understands themselves to be) is constructed within their social and cultural context [17]. An identity centred upon AN means that “fighting against AN is a difficult fight against one’s identity” [16] (p. 43). A second qualitative metasynthesis found that a key component of the treatment experience is a process of finding one’s own identity outside of the AN identity [18].

Identity shifts from the known AN identity may be met with fear and uncertainty as the person is encouraged to move towards a new and unknown identity [29] constructed in the process of recovery. The process of letting go of the dominant AN identity, for some, may be experienced as traumatic and/or associated with feelings of loss and grief [30]. This may imply that people with a lived AN experience have difficulty visualising a future recovered self without AN. They may be fearful of ‘losing the eating disorder’ as it is an important aspect of their identity and provides a sense of control [31, 32].

As such, there has been a shift towards reconceptualising recovery in AN. This includes the recovery model by Dawson et al. [33] and research by Touyz et al. [34] that focuses on improving an individual’s quality of life even with the limitations of AN [35]. A study by Touyz et al. [34] found that individuals receiving CBT-AN treatment centred on quality of life improvement showed significant improvements in reduced ED symptoms, greater quality of life and increased motivation to change. Analysis of individual’s lived experience of AN has provided support for this model, encouraging therapy to be concerned with not only reducing symptoms but with improving overall quality of life [14, 30]. Likewise, qualitative research has proposed that an important component of quality of life improvements is assisting patients address negative self-concepts and develop re-storied senses of themselves [14, 30, 36–38]. This speaks to the importance of understanding recovery in AN as a gradual reduction of the role AN plays in a person’s life and identity, supported through therapy. Furthermore, by de-emphasising the need for recovery to include a sudden ‘split’ from AN identities, individuals may experience less fear or hesitancy in engaging in treatment ongoingly.

Externalisation is one therapeutic intervention that invites the person to re-story a sense of themselves through linguistically separating a person’s identity from the problem [25, 39, 40]. Externalisation has been popular in AN treatments, including externalisation of the illness in FBT [25, 41–43]. Although intended to liberate the person from the illness identity, this practice has proven troubling for some individuals who [1] experience AN as both separate and a part of themselves [30, 38] and [2] lose their voice when others assume their experience to be the voice of the ED [44]. Furthermore, setting up the task of therapy to vanquish AN through the use of ‘contest metaphors’ [29], such as fighting the illness until it is eliminated, may lead to a diminished sense of agency and exhaustion when this does not fully eventuate for the person [29, 45]. Individuals therefore often have difficulties in resolving conflict between their sense of self and the AN identity [46, 47]. AN is conceptualised by some as both a friend and an enemy, which highlights the sense that AN both protects and provides control for the individual, yet also may lead to substantive pain and suffering [10, 13, 48].

Whilst there has been increased research interest into the question of identity and its negotiation in AN and qualitative examination of stages of change and factors contributing to recovery [30, 33, 49], there continues to exist a paucity of research that explores these processes within therapy sessions through the analysis of treatment transcripts. Whilst important knowledge has been gleaned from previous studies that have used self-report questionnaires, surveys, and semi-interviews [49–51], such data may have possible limitations in understanding individual’s more subtle and complex identity negotiations and experiences [12]. Analysis of treatment transcripts allows for identification of in-session processes whereby individuals are discussing and negotiating in-the-moment identity shifts and turning points within the therapeutic dialogue. This modality enables interest to be given to specific moments in the therapeutic process where individuals demonstrate a change in their relationship with AN and generate new identities for the future outside the dominant AN identity.

### Current study

The current study sought to explore individual’s identity negotiations during therapy sessions conducted as part of a randomised controlled trial (RCT) for Compulsive Exercise Activity Therapy (LEAP) combined with CBT for AN [7]. Analysis specifically focused on moments in the therapeutic process where individuals’ identities were dominated or defined by AN and where alternative identities to the AN identity could be generated.

## Methods

### Study Design

This qualitative study analysed in-session therapy transcripts for identity negotiations and shifts for a sub-set of the original sample RCT by Hay et al. [7] Therapy was bi-weekly for three months and weekly thereafter for 8–10 months, with follow-up at three and six-months. Treatment sessions were audiotaped initially for the purpose of checking therapy fidelity to the treatment manual. Informed consent and ethics approval were provided at the time of transcription for fidelity analyses, with extended consent gained from Western Sydney University.

LEAP is a novel therapy developed to treat compulsive exercise in individuals with AN [7, 20]. All participants engaged in the RCT received CBT-AN and half were randomised to the LEAP condition where the intervention was embedded within the first 10 sessions of CBT.

Pike, Carter & Olmsted’s CBT-AN manual [20] consists of four phases:

1. Phase 1: Orientation, engaging patients and enhancing motivation for recovery;
2. Phase 2: Core principles of CBT that consisted of a weight gain protocol, behavioural interventions and experiments, cognitive restructuring and thought records;
3. Phase 3: Schema-based CBT, affect regulation, interpersonal effectiveness (and module for those who binge eat and purge), and utilising the therapeutic relationship;
4. Phase 4: End of treatment and relapse prevention.

The LEAP component of treatment is a novel therapy developed to treat compulsive exercise in individuals with AN and integrated into each phase of the CBT-AN treatment [24, 41]. It seeks to challenge the role of compulsive exercise as a maintaining factor of AN and promotes healthy beliefs and behaviours, utilising psychoeducation, cognitive restructuring, and behavioural experiments.

In the original RCT, LEAP and CBT-AN were associated with similar reductions in ED symptoms and weight gain [7]. Further research by Rankin et al. [37] analysed in-session transcripts from a sample from the original RCT [7] for shifts in motivation to change throughout the treatment. This paper extends that research with a particular focus on qualitatively analysing participant identity negotiations and shifts early, mid- and late in the LEAP and CBT-AN treatments.

### Participants

Nine participants (1 male, 8 females, M_age_ = 26.67, SD_age_ = 5.16) from the 78 participants in the original RCT [7] in session transcripts, over three time points, were qualitative analysed. Seven participants were in the LEAP treatment group (i.e., two sessions of CBT-AN, eight sessions of LEAP embedded within CBT-AN sessions, and then 24 further sessions of CBT-AN) and an additional two participants were in the treatment as usual group (i.e., only CBT-AN sessions). Participants whose session transcripts were analysed for this study were determined by availability of previously transcribed sessions from the original RCT [7] where session recordings and transcripts were completed to analyse fidelity to the manual. The final dataset consisted of 40 in-session transcripts (approximately 40 h) from nine participants from sessions at early, mid and end points of treatment. See Table 1 for the clinical and demographic characteristics of the participants.

**Table 1:** Participants and treatment interventions

Participant	Age	Sex	Group	Transcript session numbers
Participant Z	28	Female	LEAP	2, 3, 9, 15, 32
Participant Y	28	Female	LEAP	1, 9, 13, 24, 33
Participant X	24	Female	CBT-AN	1, 5, 10, 20, 25
Participant W	36	Female	LEAP	1, 7, 14, 30
Participant V	29	Female	LEAP	1, 8, 12, 21
Participant T	20	Female	CBT-AN	1, 3, 14, 23
Participant S	23	Female	LEAP	1, 6, 13, 26
Participant R	27	Male	LEAP	1, 3, 4, 13, 22
Participant Q	20	Female	LEAP	1, 4, 13, 20

The full sample from the parent RCT were recruited from clinics and community advertising and were required to have a BMI between 14 and 18.5 and to have reported at least one exercise activity during the previous month. Exclusion criteria included a diagnosis of psychosis or bipolar disorder, high risk of suicide, medical compromise that precluded outpatient care, and a DSM-5 substance use disorder. Participants were assessed according to Body Mass Index (BMI), the Anorexia Nervosa Stages of Change Questionnaire (ANSOCQ) [52] and the Eating Disorder Examination Questionnaire (EDE-Q) [53] scores at baseline, mid-treatment, and at the end of therapy. The clinical characteristics and scores of participants are presented in Table 2.

**Table 2:** Participant clinical characteristics

	Anorexia nervosa stages of change questionnaire						Eating disorder examination questionnaire						Body mass index					
	T1	T2	T3	T4	T5	T6	T1	T2	T3	T4	T5	T6	T1	T2	T3	T4	T5	T6
Median	2.45	2.63	3.20	3.14	2.65	3.00	3.98	3.19	2.94	2.46	2.25	2.49	16.57	16.24	16.87	17.20	16.94	17.90
IQR	1.06	1.38	1.02	1.30	2.05	1.89	2.18	2.78	1.62	2.58	1.60	2.44	1.04	1.39	2.00	3.34	4.10	4.07

T1 = baseline, T2 = weeks/LEAP end; T3 = 20 weeks/mid-therapy; T4 = 34 weeks/end therapy; T5 = 3 months follow-up; TB = 6 months follow-up

*IQR* Interquartile Range

### Data analysis

Session audiotapes were transcribed verbatim, identifying material was removed and pseudonyms used for confidentiality. Analysis was conducted using six phases of an inductive thematic analysis by Braun and Clarke [54]. This analysis focused on identifying surface and latent meanings and some of the discursive material [17] participants used to negotiate their identities through a constructivist framework. Consistent with the focus of this study on key identity negotiations in a therapeutic context, the thematic analysis was informed by a constructivist philosophical positioning that understands identity as a process of (re)construction of identities with the discursive materials available to the person at the time and within a socio-cultural context [17]. Likewise, an inductive thematic analysis approach allowed for coding of therapy transcripts to generate themes around how the participants (re)negotiated and constructed a sense of identity with in a therapeutic context.

The phases of the thematic analysis [54, 55] included: familiarisation with the data, generating initial codes, searching for themes, reviewing themes, defining, and naming themes, producing the report. NVivo software was used by one coder (LH) to generate initial codes, searching for themes, reviewing themes, defining, and naming themes, producing the report. During this process, nodes were redefined and renamed. Overarching themes and subthemes were identified, defined, and given titles. These were discussed between the authors (LH, PH & JC), refined and discrepancies resolved through discussion. Exemplar quotes were presented and analysed within each subtheme. All quotations have been listed in Additional File 1: Participant Extracts.

### Reflexivity

Reflexivity refers to the process of reflecting on one’s philosophical stance and how the researchers’ lens inevitably shape the analysis of transcripts [56]. In adopting a constructivist lens and thematic analysis approach, the researchers acknowledge how pre-existing theory and experiences will impact upon positioning and interpretation of the data. This framework emphasises the importance of researcher transparency to understand the lens through which data has been interpreted and the role of language this process [17].

The first author (LH) is an Australian female completing a PhD in Clinical Psychology during the research project. She has a previous lived experience of an ED, therefore, it is possible that data analysis may have been affected by this personal lens. More specifically, this may provide advantages to data interpretation due to their insider research status [57] and ability to infer implied meanings or identify particular aspects of in-therapy transcripts to be analysed [58]. Alternatively, it is acknowledged that a lived experience background may lead to over-identification with participant experiences that were similar to their own [59]. Reflections on how this author’s lived experience may have shaped data interpretation were considered in academic supervision discussions provided by both primary and secondary supervisors Dr Janet Conti (Clinical Psychologist) and Professor Phillipa Hay (Consultant Psychiatrist). Both Dr Janet Conti and Professor Phillipa Hay are Anglo-Australian clinicians and researchers who have extensive experience providing treatment and therapy for people living with AN, including those who have recovered after 20 + years. Dr Janet Conti and Professor Phillipa Hay also specialise in the field of research and have interest in researching eating disorder treatments from the perspectives of lived experience, to inform future treatments and how these may be tailored to the unique needs and preferences of the experiencing person.

## Results

Therapy transcripts where identity negotiations were evident were analysed to inductively generate two main themes with embedded subthemes to capture the identity negotiations evident as the participants engaged in CBT-AN/LEAP or CBT-AN only.

1. Troubled Identities
  1. Conflicted Identities
  2. Identities as Othered
2. Rebuilding Identities
  Theme 2a: Shifting Relationship with Oneself
  Theme 2b: Building Life and Identities Outside of the AN Identity

**Fig. 1:**
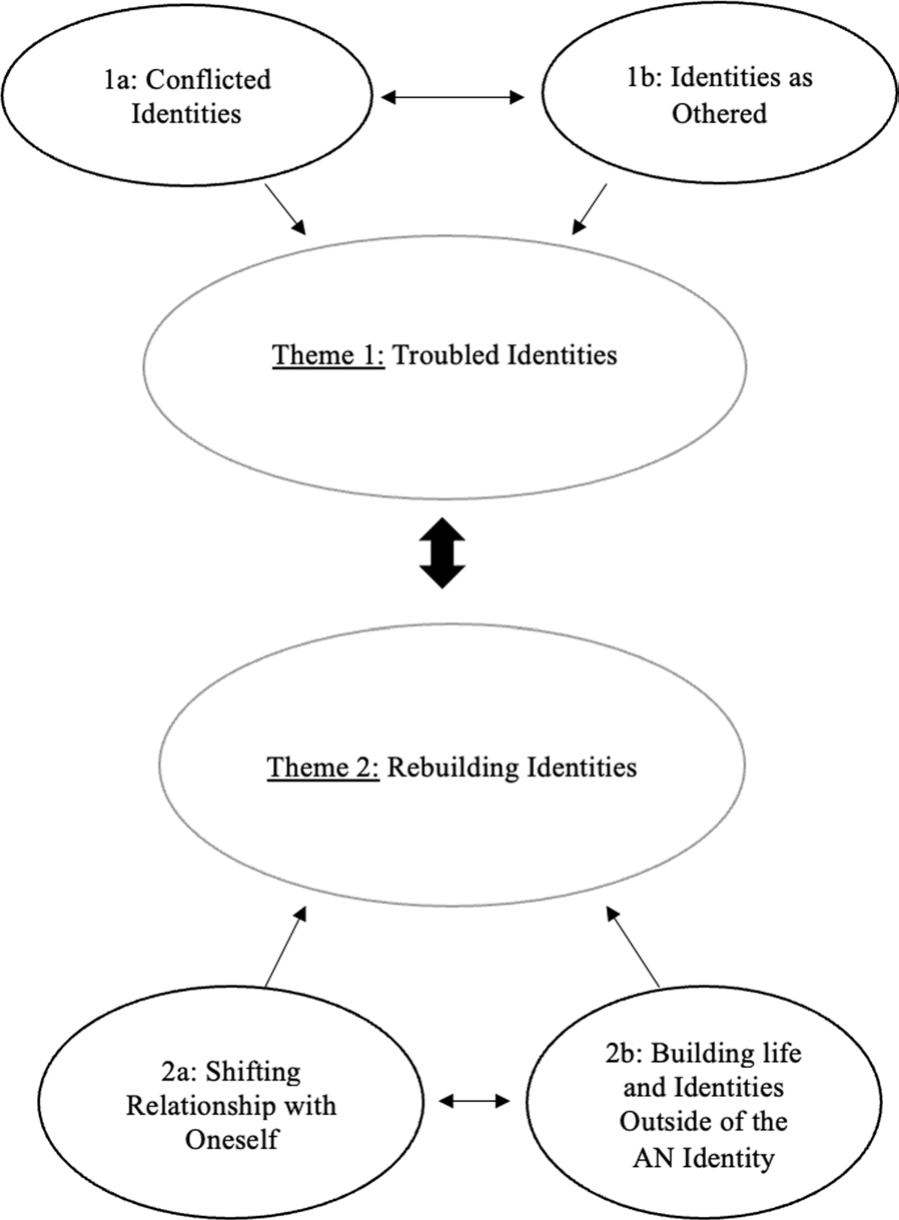
Thematic map

The inter-relationship between these themes and subthemes (Fig. 1) was recursive with the person returning to the AN identity, albeit less frequently, over time. See Additional File 1 for all participant extracts.

### Theme 1: troubled identities

When therapy sessions focused on identity talk, the participants’ identities were troubled by the AN identity and what this meant to them as a person. At these points in therapy conversations, the AN identity was both conflicted (Theme 1a) and othering (Theme 1b) of the person with a lived experience.

### Theme 1a: conflicted identities

The range of identity positions taken up by participants when talking about the AN experience over the course of treatment varied and had implications for the extent by which the AN identity was conflicted. This extended to a struggle in navigating a sense of identity both with and without the presence of AN.

#### Early therapy sessions

Within early therapy sessions, participants took up a variety of conflicted identity conclusions. For example, Participant Y concluded that they *‘…felt really weak… I need to stop being such a baby’* (S1), and Participant V that they felt *‘…really dirty’* (S1). These negative identity conclusions appeared emblematic of a broader struggle for participants to live with and accept themselves. Furthermore, participants negotiated to what extent their identity was interwoven with AN, including questioning whether AN was them, a part of them, or separate to them. For Participant Y, the troubling identities generated by AN led to a loss of agency to AN whereby they became *‘…dissociated from a rational person and you just have to act that out because you’re possessed by it’* (S1). This contributed to a sense of separation from the self and disintegration of Participant Y’s identity outside of the AN identity. The impact of this was a picture of a divided sense of self that was mapped along the fault lines of the AN experience.

Participants S and V resisted the idea that the ED was separate from their sense of selves (“separation externalisation”) [60] (p. 139). Instead, Participant S took up the position that ‘There are definitely two sides of me, the rational side of me and the ED. I really do not feel like there are two different voices or things like that’ (S1). This suggested that whilst Participant S experienced different parts of herself not occupied by the ED, there were also aspects of her identity that included AN that were positioned as “two sides of me”. For Participant V, AN was not positioned directly as separate to the self, however, they drew on externalised language when explaining how ‘it [AN] has control’ (S3). Alternatively, for Participant Q, her own research into AN led her to position herself outside the AN diagnostic category, despite also seeking treatment, i.e., ‘I research all that stuff… I just don’t see myself as someone who has it’ (S1).

The conflicted nature of AN was also evident at points when participants argued that AN was protective. Participant Z noted that the pain of compulsive exercise was experienced to her as a *‘good thing’* (S2), and Participant R described AN as *‘familiar’* and *‘secure’* (S3). Within the same stretch of text, an antithetical position was taken up where participants appeared to be arguing within themselves against a life dominated by AN, which was described by Participant Z as *‘running myself into the ground’* (S2) and *‘destructive’* (S9) by Participant R. At this point in therapy, these discussions were often dialectical in nature where participants appeared to attempt to resolve these seemingly conflicting positions.

#### Mid-therapy sessions

Although there appeared to be less AN-dominated negative self-concepts expressed during mid-therapy sessions, participants continued to negotiate identities that were shaped by societal values of the thin body (i.e., thin). Participants S and Y described a sense of failing to meet the cultural ideals of the thin and exercise shaped body as leading them to see themselves as *‘lazy’* (S6; Participant S) and *‘not even as good as any of these people’* (S9; Participant Y). Implicit in these extracts was a moral discourse whereby participants’ sense of self-worth was measured by their engagement in exercise and attainment of the thin body.

Likewise, participants appeared to enter more nuanced and complex discussions regarding their experience of AN as being both separate and intertwined with the self. Participants’ positioning on this question had implications for the extent to which they experienced personal agency over AN. This included discussions on whether they perceived AN to be a form of control, to be controllable, and/or in control of them. The nuanced nature of these identity negotiations was exemplified by Participant V who stated, *‘I don’t know if that’s all driven by the ED, or it could just be my personality’* (S12). Within this quote, Participant V first took up a position that separated the ED from the self, whereby this meant the ED took on a life of its own. They then took up a conflicting position that internalised the ED as part of her personality. Both positions had the effect of diminishing the participant’s sense of personal agency. This dialectic around the question of personal agency was also evident for Participant X, who first described AN as having total control over their life—*‘It dictates everything’* (S10). Alternatively, later in the same session, Participant X reflected that regardless of this they *‘…don’t want to let it [the ED] go. I just cannot let it go’* (S10).

For Participants V and X at this point in therapy, the uncertainty of living with the self without AN appeared overwhelming and a dual relationship with AN emerged. Participant V highlighted the conflictual nature of the AN experience with the recognition that AN was causing her distress but also that she perceived that AN *‘…helps me to live with myself’* (S12). Likewise, Participant X expressed a fear that *‘pain and stuff’* (S10) might come to the forefront of her life in the absence of AN. Implicit in this was a function of AN to push back past painful experiences which made it challenging to ‘let go’. Moreover, these difficulties in having agency to let go of AN were implicit in Participant Z’s desire for the AN to *‘go away’* (S15). Participant Y highlighted further complexities in navigating ideas of self-agency or control over AN, evident in her concern that *‘As I try to control the [restricting] more, I wonder if I’m going to try and be more controlling of other parts of my life’* (S9). This speaks to a conflictual relationship with agency and control, in particular Participant Y’s concern that increased control over AN symptoms may lead to her wanting to control other aspects of her life more, perhaps in unhealthy ways.

#### Late therapy sessions

The struggle with conflicted identities related to the AN experience was ongoing for some participants in later therapy sessions. Participant X continued to draw on a moral discourse that meant her hopes and desires were confined to the attainment of the thin body—*‘Everything you want is to be skinny’* (S20). Implicit in this quote is Participant X’s previously expressed struggle to live with themselves should they not be thin. Additionally, Participant R described AN as a protective layer to a ‘fake’ identity that lay underneath—*‘… people will see through me… Like I’m a fake’* (S22). The vision of recovery for Participant R was one of subscribing to societal norms and *‘…trying to do everything I could to mimic what everyone else was doing to fit in. To not look like a fraud’* (S22). Without AN, Participant R imagined they would be left with a fraudulent identity that would eventually be exposed to others. This highlighted the difficulties experienced by this participant in letting go of AN that were intertwined with an underlying impoverished self-concept.

Participants’ accounts in later therapy sessions continued to highlight the complex ongoing identity negotiations in relation to AN, in particular the extent to which each experienced themselves as having personal agency over AN. For example, in positioning the ED as separate to herself, Participant W’s narrative that she had not *‘…been in control for a long time’* and that *‘The ED has been in control’* (S30) continued to be reinforced. On the other hand, Participant R’s account of her identity in relation to AN was internally conflicted between a part of her that *‘…knew that I had to put on weight and I had to change my behaviour’* and another part that felt making these changes was *‘…connected to losing control’* (S22). Participant S’ identity in relation to the AN experience shifted later in therapy, which was evidenced by her comment that it [AN] was no longer *‘…the same anorexia I was back then’* (S26). This suggests that while AN continued to have a role in her self-conceptualisation, this role had shifted and her personal agency over AN had increased, with a sense that Participant S was standing for herself at this point in the therapy—*‘I definitely would do something to correct that [if ED symptoms returned] because I know I am there for me’* (S26). Whilst participants continued to grapple with a sense of personal agency in their relationship with AN, what was also evident was their increasing insight into their internal identity negotiations and considerations of how they might like their relationship with AN to be different.

### Theme 1b: identities as othered

All but one of the participants spoke about a sense of feeling othered by the AN experience through (i) the sense of isolation from friends and family relationships who struggled to understand their experience, and (ii) being positioned as outside the boundary of normality.

#### Early therapy sessions

Early session transcripts indicated that participants’ experiences of AN were often associated with a sense of being othered and leading to isolated identities. AN was talked about as contributing to a sense of alienation and separation from others in their life, including their loves ones. Participant X felt as though their family members did not understand their experience of an ED, stating, *‘…he does not get my own issues… he does not get my point’* (S1). Implicit in this was the experience of an isolated identity generated by the ED that others could not access. A unique experience of an isolated identity was further described by Participant S who had a sister also struggling with AN. Comparisons with her sister’s perceived recovery contributed to strain in her family relationships where she did *‘…not want to prove myself to people who do not realise how difficult it is to maintain weight’* (S6). When Participant W reflected on how the AN had contributed to the impacts of her actions on others, the most available identity position reduced her sense of herself as a person to *‘… short tempered and impatient and just a screaming mother’* (S1). A further issue of blame was raised by Participant V who spoke of her family members defending their identities against blame for the AN, i.e., *‘They get really defensive. They think it’s all their fault’* (S1).

Early therapy sessions also explored the impact uptake of discourses of normality had in shaping participants’ identities as other than ‘normal’. Participant X argued that although *‘Every day I wanted to work out, I never wanted to be crazy’* (S1). This indicated that in taking up the AN identity, there were limited identity positions available outside of a pathologized and disorganised identity. On the other hand, Participant Z described a resistance to therapy that she experienced as *‘… trying to normalise me’* (S2), including an over focus on food and journaling her meals, stating, *“It does not feel normal. Normal people do not write down food’.* (S2). She instead argued that *‘food is not the issue’* and that she needed treatment to focus on the issues that were relevant to her. Participant V also positioned herself outside normality when noting her *‘best method’* for recovery was to ‘*line myself up with an engagement with someone… And so that way I go to control myself to being normal’* (S8). Implicit in Participant V’s account was a fragmented identity outside of AN where normality was something to be controlled into by mirroring the eating behaviours of others not suffering from an ED. The inherent challenges of defining so called normality meant that this was construed as a vague and distant goal that could not be well described.

#### Mid-therapy sessions

Discussions of relational difficulties and how these contributed to isolated participant identities continued in mid-therapy transcripts. Participant X described AN as affecting *‘…your relationships and it puts a strain on things… [the ED] makes you have more arguments with people more often because they don’t understand you… only you understand yourself and it’s just easier to be alone’* (S10). For this participant, the strain caused by AN and profound feelings of being misunderstood led to a resignation towards ongoing solitary identities (i.e., *‘easier to be alone’*). This sense of isolation contributed to an identity as othered as exemplified in her description: *‘…instantly feel[ing] bitter towards really thin people, because I wish I was them’* (S10). For Participant T, isolated identities were driven by not wanting others to *‘see me eating’* (S14), which limited her capacity to socialise and connect with others.

Participants continued to be positioned by discourses of normality in mid-therapy transcripts. Participant V described a process of *‘trying to adopt more of a normal person’s perspective in arguing with the disorder’ and that ‘…because there were other people around, it was probably more important that I looked or appeared to be normal’* (S12). These extracts show how through positioning herself outside the boundary of normality, becoming ‘normal’ was construed as required for the motivation or goal for recovery. Likewise, these statements positioned normality as something required to be socially accepted by or connected to others. Participant Y indicated that she felt regular exercise was fundamental to her self-worth and ability to meet ‘normal’ standards—*‘If I’m not doing exercise then I’m not even as good as any of these people. I’m like in a different level of society’* (S9). Participant W’s account exemplified an internal dialogue of shifting positions between seeing herself as *‘normal’, ‘huge’,* and then *‘not anorexic anymore’,* i.e., *‘I feel normal. I feel huge really […] I’m 44kgs, that’s a normal person weight, not an anorexic weight. I’m not anorexic anymore […] I’m seeking treatment but I’m not anorexic’* (S14). The implications of this were a conceptualisation that a person’s weight could fall inside or outside the bounds of normalcy and the implications (perceived or real) for her legitimacy to continue to engage in ED treatment should she or her weight be ‘normal’.

#### Late therapy sessions

Ongoing fears of being judged or rejected by others were evident in therapeutic conversations in later sessions, contributing to a continued sense of isolated identities. Participant R expressed concerns about *‘worry[ing] or burden[ing]’* others, and *‘the chance that the new people won’t like me or will reject me’* (S22). Likewise, Participant Z disclosed that they were *‘…normally stressed about if my new friends are judging me…’* (S32). For Participant W, isolated identities generated by AN continued to be exacerbated by a sense that others *‘… do not understand what it [AN] is’* (S30). Overall, the extracts from end-of-therapy sessions highlighted the enduring sense of identity as othered that persisted and was exacerbated by struggles to feel understood and emotionally safe in relationships, fears of criticism, and the sense of oneself as not normal.

### Theme 2: rebuilding identities

How participants depicted their shifts in their relationship with AN had implications for who they understood themselves to be and how they conceptualised recovery. These shifts included (a) shifting relationship with oneself and (b) the building of life and identities outside of the AN identity through finding previous identities lost in the AN experience.

### Theme 2a: shifting relationship with oneself

Most of the participants spoke at some point across the three therapy sessions about what recovery meant to them individually and their vision of themselves as ‘recovered’. The nature of these identity negotiations fluctuated over treatment.

#### Early therapy sessions

Within early therapy sessions, participants were able to identify hopes and dreams for recovery. Rather than characterised by a reduction of symptoms or increased weight, recovery at this point in the therapy was spoken about holistically as an embodied shifting relationship with oneself. This included Participant Y who identified that her *‘…greater goal would be… just relaxing into the way I am a little more’* (S1). Participant R conceptualised recovery as allowing the self to *‘accept the practical and rational reasons for taking it easy sometimes’* and *‘allowing the body to recuperate’* (S4). This greater connectedness to the self was also envisioned as an improved responsiveness to the body. Likewise, Participant X discussed the importance of recovery being about having a *‘happy and healthy mind’*, rather than a sole focus on weight or symptom reduction—*‘There is a difference between a healthy mind and a healthy body. My body wants to be healthy and happy, but where is my healthy and happy mind?’* (S1). For Participant Q, *‘self-esteem [was] the main thing’* (S1) involved in recovery. Implicit in these extracts were participants’ abilities to use complex and nuanced descriptions of recovery that was fundamentally related to a shift in relationship with the self. Simultaneously, participants experience of this connection with the self was that it had been disrupted by the AN experience.

#### Mid- and late therapy sessions

In transitioning from early therapy to mid- and late therapy sessions, discussions conceptualising recovery appeared to become more troubling for participants. Where once able to clearly identify descriptions of recovery from AN, participants appeared to struggle to generate these preferred ways of being, particularly in the context of a medicalised version of recovery where the illness is assumed to be gone. Participants experienced difficulties imagining their life on the terms of this medical discourse, where the only available identity slots are either sick or recovered. For Participant Z, this was expressed in her statement, *‘I don’t know what recovered is like, I don’t know what it feels like’* (S15). Likewise, Participant V considered that *‘Maybe if I got better at this… maybe the stronger I believe this, the more likely it’s going to “beat” the ED itself’* (S21). These extracts demonstrated the emphasis participants placed on their hopes for eradicating AN from their life, and the sense of a distant and undefined sense of identity that came with these hopes.

### Theme 2b: building life and identities outside of the AN identity

Whilst some participants struggled to conceptualise recovery and what this meant to them, there were stretches in the therapy sessions over time for the majority of participants where shifts in their relationship with AN appeared possible. This was through a shift in focus from eliminating the AN symptoms to building the sort of life and identities they hoped for.

#### Early sessions

Early in therapy sessions, participants found it difficult to engage with conversations around building life and identities outside of AN. Instead, therapeutic dialogue focused more on exploring the role, function, and impacts of AN on the person’s life. During their first therapy session, Participant X, however, was able to identify aspects of her life that she wanted to reclaim from the ED and that spoke to her values and what mattered to her—*‘… I used to play a lot of music and write things… I want to go back to only keeping myself busy with things that make me happy and not the ED […] I really like it that I can slow down, sit with my kids, you know this has really started to come back’ (*S1).

#### Mid- and late therapy sessions

Therapeutic conversations throughout mid- and late therapy sessions saw participants more frequently explore how shifts in their relationship with AN would impact their identities and ability to rebuild their lives. For Participant S, rebuilding life was an acknowledgement that *‘I can add another chapter in my life’* (S13) which she described as *‘very exciting’.* The language used by Participant S in this quote of *‘I can’* highlighted an increased sense of agency and ownership over the direction of her life. Likewise, Participant W described recovery as an increased focus on the rest of her life outside the disorder and ‘letting go’ of AN—*‘I just want to start to be able to not think about it and want to get on with my life without giving it focus. I want to start letting go’* (S14). Central to Participant W’s preferred sense of identity were her values and re-establishing a connection with herself and others with an externalisation of AN through the use of ‘it’. This was also evidenced by a journal extract that Participant W read out to her therapist which depicted a connection with a valued sense of self—*‘Calm and happy, nice, patient, kind, logical, healed, transmit health, compassionate, beautifully strong and fit, relationships, healthy, balanced, happy kids, help others, freedom, picnics, going out for dinners, loving self, socially free and liberated’* (S14). This journal had scope to provide Participant W with a map to navigate her identity as she migrated from a life and identity dominated by AN. Nearing the conclusion of treatment, Participant Z indicated that she felt she was *‘Working together for the goals… and crossing them and building a future’* and that *‘it feels so nice to be here’* (S32). This conceptualisation of recovery was linked to a sense of rebuilding a life outside of AN, characterised by a greater knowledge of, working towards, and achieving her personal goals. Likewise, Participant S described identity shifts in terms of increased independence and confidence, in addition to taking back control over her choices—*‘… a lot more independence […] You have more control over how you manage meals and everything’* [26].

## Discussion

This study explored identity negotiations of participants in the LEAP treatment for AN [7] that focused on targeting compulsive exercise behaviours in addition to the core CBT-AN components [20]. Through qualitative analysis of in-session transcripts, two key themes pertaining to identity negotiations throughout therapy were generated: [1] AN experiences generated troubled identities, and [2] rebuilding identities and life outside of the AN identity. The identity negotiations evident in the therapeutic conversations aligned with the key components of the CBT-AN intervention, including addressing [1] the characterisation of oneself as ‘an anorexic’ and the over-evaluation of weight and shape in one’s self-concept, and [2] working towards diversification of roles and activities to broaden and enhance one’s self-concept [20]. Exploration of key identity negotiations indicated that early therapy sessions centred predominantly on conflicted identities and the sense of othering that were generated through the AN experience. Identity negotiations were therefore a recursive process going between the AN identity and alternative self-concepts that included hopes for recovery and their future, with more talk related to the building of new identities and the struggles to imagine future recovered selves in the mid to later therapy sessions. Thus, the findings of this study support the potential for CBT-AN [20] to therapeutically address self-concept over the course of therapy.

During early therapy sessions, participants explored the troubled impact AN had on their self-concept and negative identity conclusions which were constitutive of a broader struggle to live with and accept themselves. These identity struggles impacted upon participants’ sense of agency, with AN conceptualised as a form of control over their lives. In early and mid-therapy, participants also engaged in dialectic conversations where they appeared conflicted about whether to invest in a life with or without AN. Participants’ struggles with the AN identity included questioning whether AN was separate and/or intertwined with the self, which had impacts upon their ability to reconnect with a sense of personal agency, i.e., who is in control, me or the AN? Within these therapeutic conversations where AN was separated from their identity, there was a tendency for the person to perceive themselves to be without agency in the face of overwhelming influence of the AN. Overall, the participants in this study appeared to struggle with the practice of separating their identity from the AN (or separation externalisation [38] because this did not account for the times that they perceived the AN to be part of their identity. Alternatively, once the AN was separated from the self, it risked being perceived as a powerful force over which they did not have control.

Within mid-therapy sessions, participants continued to struggle with identity negotiations shaped by societal values of the ideal thin body, although appeared to engage in more nuanced and complex therapeutic conversations which explored the degree to which AN could be experienced as separate from the self. For many participants, this included further discussions about personal agency and to what extent AN was a form of control, controllable, and/or in control of them.

For some participants, struggles with their sense of self and the role of AN in controlling their identity and life continued in late stages of therapy, however, a shift was seen in participants’ development of insight and ability to consider how they might like their relationship with AN to be different. At this stage of treatment, the intervention had moved to include more Schema-based CBT techniques, which contributed to rich conversations surrounding the development of a broader self-concept [20] and in some instances, the generation of identities outside of the AN identity.

Participants also engaged in identity negotiations in therapy by grappling with discourses of normality which further impacted on to what extent they felt othered, leading to a sense of isolation and marginalisation in the AN experience. Throughout early and mid-therapy sessions, in taking up the AN identity, participants were positioned outside the boundary of normalcy [47, 61]. From this place, it was also evident that the AN identity had limited identity positions for the participants to see themselves outside of a pathologized and disordered identity. This is consistent with previous qualitative research that has explored participant experiences of AN treatment, where individuals discussed ideas of what behaviours and feelings constituted normality and how this might impact on their ability to negotiate an identity outside of the ED [62, 63]. Likewise, one participant in the current study described previous treatments as having an over focus on normalisation of eating behaviour, thus reinforcing a disordered self-concept heavily aligned with the AN identity. Previous research by LaMarre et al. [64] indicated that individuals receiving treatment for EDs often felt that achieving normalcy was spoken about primarily regarding nutrition and weight, and positioned as a crucial factor in future recovery. This was thought to be at the expense of opportunities to define normality and explore its role in how individuals conceptualise recovery, and posed questions about what is meant by ‘normal’ in a westernised context where the thin body is idolised. Further, this participant’s experiences of previous treatment were reflective of research by Malson et al. [65], where participants expressed that treatment for EDs often involved ‘hyper-disciplined micromanagement of the body’ (p. 5) that are not usually considered ‘normal’ outside of treatment contexts.

Although participants frequently talked about themselves as outside the bounds of normalcy, they also struggled to articulate what so called normality was. Central to this troubled identity is the limitations of a disorder discourse from which Gergen and McNamee [47] have asked: ‘When does a person who is “disordered” become “undisordered” and who decides?’. As therapy progressed, conversations appeared to focus less on the concept of normality as participants shifted towards consideration of more flexible future self-concepts outside of the AN vs. normality discourse.

Another way that participants experienced themselves as othered was through the struggle to feel understood by others that they talked about particularly in early sessions. Participants engaged in identity negotiations as they talked about the impact of AN on their relationships, including a sense of oneself as isolated, mad, and sick. This is consistent with theories that propose that not only are social environments that value thinness and body shape capable of further maintaining AN pathology, but that individuals who experience AN carry the further burden of vulnerability to the opinions of others [11, 66]. Relational difficulties and their contribution to participant’s isolated identities continued to be discussed in mid- and late- therapy sessions, however, deeper conversations regarding driving forces to this isolation (i.e., lack of emotional safety and fears of criticism and rejection) were also generated in therapy sessions.

In-session identity negotiations also had implications for how participants envisioned their hopes for recovery, including conceptualisations of their relationship with self, and rebuilding their life and identities. In early therapy sessions, participants’ hopes and dreams for the future appeared more easily identified and included a wish for a holistic relationship with the self, where the self has an improved responsiveness to the body. Participants tended to identify recovery as a greater contentedness with self and described this as beyond the reduction of physical symptoms. This is consistent with research that suggests the construction of recovery from AN as being linked to the absence of symptoms has been a limited one for many individuals [46, 67]. Throughout mid and late-therapy sessions, these discussions appeared to become more complex for participants. Whilst participants discussed their desire to eradicate AN from their life, they also wrestled with the distant and undefined sense of self this brought due to a complex and partially externalised identity from AN. This is reminiscent of research by Malson et al. [68] where participants simultaneously desired recovery yet also struggled to envision future recovered selves, an experience that was termed ‘Un/imaginable future selves’. When recovery was conceptualised by participants, they tended to depict this with the use of ‘contest metaphors’ that posed AN as something to be fought against and eliminated [45, 69]. White [29, 45] has cautioned against such battle metaphors as they set up the task of therapy to be adversarial, which may be troubling for individuals who conceptualise AN as in some way ‘part of themselves’ and lead to a sense of exhaustion if and when aspects of the AN experience are unable to be eliminated. The discussions participants had in this study were reminiscent of participants in a qualitative study by Conti et al. [36] whose relationship with AN overtime resembled a shifting relationship with AN and a reclaiming of life and identity from the AN. That is, in contrast to efforts to separate AN from their identity with the aim to vanquish it from their life.

Participants struggled to engage with conversations around building life and identities in early therapy sessions, with discussions focusing on the impacts on AN on the person’s life. In mid-therapy sessions, participants began to process how shifts in their relationships with AN would impact their abilities to take back their lives. Participants also described experiences of an increased sense of agency and ownership over their values and goals. This led to identification of aspects of their lives they wished to reclaim, including those that spoke to an imagined future with greater connection to a valued sense of self and with others,. In late-therapy sessions, participants were better able to imagine building their lives outside of AN, which lead to improved abilities and confidence to live out new identities. An imagined future was made possible in these stretches of text and was less troubled than when participants imagined their life with AN eliminated. This provides support for the recovery model [33] where recovery does not necessitate total symptom remission but is instead focused on improving one’s quality of life by living a ‘satisfying, hopeful, and contributing life even with limitations caused by the illness’ [70].

### Limitations and strengths

The strengths of this study are the in-session nature of the qualitative data that provided a unique dataset to explore longitudinal identity negotiations in a therapeutic context. Likewise, use of a secondary dataset was considered to be an ethical use of data already collected to inform further understandings of the experiences of individuals with AN. This decreased participant burden by not recruiting a new sample of participants to engage in intervention and by not requiring participants to detail experiences already described in-session, such as in the form of an end of treatment interview. Furthermore, the author regularly engaged in reflection with the co-authors (JC and PH) and discussed theme development so that consensus from the researchers was reached.

The limitations of this research include that transcript analysis for this study and selection was based on pre-existing data, which yielded an uneven number of participants from the CBT-AN and the LEAP adjunct conditions. Likewise, the transcripts were only included of the participants who completed the intervention. More research is needed into the identity negotiations of those who do not complete therapy as these individuals are at greater risk of progressing on to severe and enduring AN. Additionally, the current study was a secondary analysis from an RCT by Hay et al. [7] and a primary study of interviews with people about their own perceptions of key identity negotiations in treatment would further such research.

### Clinical implications and future research

The findings of this research suggest that identity negotiations around the AN identity are key in the AN experience. Future treatments may consider broadening their focus on these, particularly on processes that generate identities outside of the AN identity to rebuild preferred identities [30, 67, 68]. This study demonstrates that during the LEAP/CBT-AN intervention, participants were actively engaged in identity negotiations, however, there were also opportunities to explore these negotiations further not taken by the therapist to ensure fidelity to the manual. Whilst further quantitative research could examine the hypothesis that the two interventions would impact on the dialogue related to identity negotiations, no themes emerged that suggested experiencing one or the other intervention group influenced this dialogue.

The following practices may extend therapeutic conversations to more comprehensively explore identity negotiations (including rebuilding a sense of self outside the ‘anorexic’ (AN) identity [11]. These interventions would benefit from further research:

- Extending externalising conversations to explore both the influence of AN on a person’s life and identity AND the influence of the person over the AN. Michael White termed this practice ‘relative influence questioning’ [48], which is the second side of externalising conversations that is designed to explore openings where the person has experienced a sense of agency over the AN. These openings are frequently lost when AN is experienced as a powerful force over which the person perceives themselves to have no control.
- Inviting re-authoring conversations that are central to the practice of narrative therapy [39, 45] to explore how people reclaim and strengthen past identities that have been lost to the AN identity, rather than focusing mainly on current and future identities to build new identities.
- Use of relational externalisation where the therapeutic focus is on reclaiming life from the AN and re-building new identities rather than seeking to separate the self from the illness in order to eliminate it [36, 71].
- Likewise, this study highlights how the use of adversarial metaphors that position AN as something to be fought against may be troubled and contribute to a sense of exhaustion when this does not eventuate [45]. Instead, relational metaphors, such as ‘reclaiming identity/life’ may be helpful to describe a sense of salvaging life from AN and re-authoring renewed and preferred identities [29, 30].

Extending therapeutic conversations from building new identities to rebuilding new-old identities [72] would benefit from further research to understand whether this leads to more sustainable change as a person seeks to rebuild their life and identity in the wake of AN and its effects.

## Conclusions

The exploration of identity negotiations during the course of treatment for AN is a relatively new area of research [27, 73]. Despite this, recent research suggests that difficulties with these identity negotiations may present as a significant barrier to treatment and recovery [32]. The study has demonstrated that a CBT-AN intervention (with or without LEAP) went some way in exploring participant identity negotiations, particularly in separating the AN from the person’s self-concept and in the building of new identities outside of the AN identity.

There is a need to explore how these identity negotiations might be further extended through therapeutic conversations that more specifically focus on rebuilding past identities that have been lost to the AN identity, in addition to the more usual CBT-Schema Therapy practice of building new roles and activities to enhance self-concept. Further research is needed to consider implications for integrating such findings into therapeutic interventions.

### Supplementary Information

**Additional file 1.** Participant extracts

## Data Availability

All data generated or analysed during this study are included in this published article and its supplementary additional files.

## Abbreviations

ED: Eating disorder
AN: Anorexia nervosa
CBT: Cognitive behavioural therapy
LEAP: Compulsive exercise activity therapy
RCT: Randomised control trial
BMI: Body mass index
ANSOCQ: Anorexia Nervosa Stages of Change Questionnaire
EDE-Q: Eating Disorder Examination Questionnaire
