# Supplementary material for: Key-in-session identity negotiations in a first line treatment for adult anorexia nervosa
Source: J Eat Disord. 2024 Jan 31;12:20. doi: 10.1186/s40337-024-00979-0 (PMC10832269; doi:10.1186/s40337-024-00979-0)
Supplement: Supplementary file 1 — Additional file 1. Participant extracts [file 40337_2024_979_MOESM1_ESM.docx]

**Additional File 1: Participant Extracts**

**Theme 1: Troubled Identities**

| **Subtheme** | **Participant Extract (Session Number)** |
| --- | --- |
| Theme 1a: Conflicted Identities | EXTRACTS 1 (early sessions):  Participant Y: I sort of felt bad and guilty and sort of not sticking to my routine. So weak, yeah I felt really weak… It’s fine, I mean, I need to stop being such a baby. (S1).  Participant V: I feel like I become dissociated from a rational person and you just have to act that out because you’re possessed by it […] I can tell you straight away that like the imaginary fat droplets… even if your size doesn’t change at all, I can feel the imaginary fat particles being stuck into you […] you feel really dirty (S1).  Participant V: It’s not a separate entity or anything, it’s just that it has control […] I think it [the eating disorder] probably had a little control but I was able to exert more control. (S1).  Participant V: I’m not sure if I could make such a clear distinction between the disorder and myself but I can make a distinction between how I think it’s ok for other people to do things but not ok for me. (S8).  Participant S: You know like I can control my weight but I can’t control my life. (S1).  Participant S: It feels sort of part of your identity as well. I don’t know whether… the eating disorder was spoken of in terms of how it can affect us instead of just external effects. (S1).  Participant S: I have read that they [others with ED] sort of have external views of the ED […] I do not really like that […] There are definitely two sides of me, the rational side of me and the ED. I really do not feel like there are two different voices or things like that. (S1).  Participant S: Obviously there is an anorexic side, which sees it as a failure, but then there is a rational person (S6).  Participant S: … I suppose for me lazy is… not doing some sort of exercise during the day. (S6).  Participant Q: I don’t know… I research all that stuff, and I, you know, I just don’t see myself as someone who has it (S1).  Participant Z: I like running myself into the ground because the pain is kind of a good thing (S2).  Participant R: I can see that it’s destructive, even though it plays such a strong role – it’s, I can see that it’s destructive. Um, on a lot of levels […] On the other hand, it’s become familiar, it’s sort of safe and secure and I know that if I follow it, I feel satisfaction. (S3).  Participant X: What I was trying to do obviously is put on weight, because I know that I have to put on weight, what I can’t do is accept it. Once I see it going on, once I see that number going up and I cannot accept it, I have to do something about it to make it go back down. (S1).  EXTRACTS 2 (mid sessions):  Participant Y: It’s that thing… thin people are, like being thin is the ultimate goal, so thin people are the most successful, worthwhile people […] Yeah, cause anything that helps you get thinner is good and anything that doesn’t is bad […] If I’m not doing exercise than I’m not even as good as any of these people. I’m like in a different level of society […] If I don’t exercise it makes me feel unsuccessful and unworthy, yeah it makes me feel lazy, like I often call myself lazy all the time. I actually consider myself to be a lazy person. (S9).  Participant Y… As I try to control the [restricting] more, I wonder if I’m going to try and be more controlling of other parts of my life. (S9).  Participant V: I don’t know if that’s all driven by the ED or it could just be my personality […] rationally, the argument against outweighs the argument for… but the argument for, it helps me to live with myself. (S12).  Participant V: In my mind if I was listening to the eating disorder, it would tell me it’s my fault […] They’re [the information given by ED] not true. That it just manifests itself because it helps to sustain the existing. (S12).  Participant X: It does change a lot of things. It dictates everything. It tells you what to do… what you eat, what you wear, this and that, so yeah it dictates a lot. (S10).  Participant X: I don’t feel underweight, I feel pretty big. I don’t know, I don’t want to go there now. I think I am doing enough. I cannot handle thinking that. (S10).  Participant X: … what I feel is some sort of conflict but I just don’t know, I have a problem with knowing when to stop. (S10).  Participant X: I feel comfort in starvation… I don’t know if I still do, ‘cause I haven’t starved in a long time. But I know that if ever something goes wrong, I would find comfort in that and I am taking control through that. (S10).  Therapist: … what is holding you back? Participant X: I just don’t feel strong enough to do it now. I just cannot in my mind because I feel like I have suffered lots of pain and stuff. I don’t want to let it go. I just cannot let go… (S10).  Participant Z: I feel like I really can’t be bothered having an ED… I know there is definitely times it is just there and I think I wish it would go away (S15).  Participant W: I still want a little bit of recognition [for efforts in losing weight], it’s how it is because of the perfectionism. (S14).  EXTRACTS 3 (late sessions):  Participant X: Everything you want is to be skinny. (S20).  Participant X: But I was trying not to feel guilty […] Every time I put back on weight that time is back where it all began… like I am turned by so much to sin, to so much guilt… (S20).  Participant W: I haven’t been in control for a long time… The ED has been in control. (S30).  Participant R: On the one hand, I knew that I had to put on weight and I had to change my behaviour, but on the other hand, I was still sort of fighting against it because I wanted to. I felt like, changing my behaviour was connected to losing control. (S22).  Participant R: I believe that people will see through me… Like I’m a fake, I’m putting on a persona […] People will realise that… there’s not much substance behind what I’m doing […] Like they will see that really I, I’m just sort of putting on this mask or persona to fit in with whatever is going on… pretending to be like everyone else […] I feel like other people know the drill. They know what to do. But I… try to sort of follow along or pretend… […] “I’d be trying to do everything I could to mimic what everyone else was doing to fit in. To not look like a fraud, sort of thing. (S22).  Participant S: I definitely would do something to correct that because I know I am there for me, it’s not the same sort of anorexia I was back then. (S26). |
| Theme 1b: Identities as Othered | EXTRACTS 4 (early sessions):  Participant X:… he does not get my own issues, like always he just sees and waits, does not get my point… He says oh you are starving, you are underweight. (S1).  Participant X: Every day I wanted to work out, but I never wanted to be crazy. (S1).  Participant X: I was thinking really questioning and watching them, like someone who eats a lot for me… it was like just grab it and then I don’t know, it is not my right, but you know it is the thing in my mind that drives me crazy, and my dad is fat and I think ‘oh he should not be doing that to himself’. (S1).  Participant S: She [sister with ED] would say she had recovered but she has not, she needs to gain weight, she’s still got a long way to go […] Therapist: So you and your sister are competitive in a way? Participant S: Yeah. (S1).  Participant S: Yeah like with my dad, he used to be like why can’t you just eat, it’s so simple […] You get that kind of people [asking] why you can’t eat. (S1).  Participant S: Yeah for a long time I stopped seeing a lot of my friends because it was a bit awkward and I had so dramatically lost weight […] It definitely creates some sort of tension, it drives a wedge between some relatives and it can create sort of awkwardness. (S1).  Participant S: I just do not want to prove myself to people who do not realise how difficult it is to maintain weight. (S6).  Participant W: I’m always angry and yelling and screaming and that’s gotten worse overtime. I’m short tempered and impatient and just a screaming mother. (S1).  Participant V: They get really defensive. They think it’s all their fault... You know, if you just ate all that food and kept it down you’ll be so healthy. And like, you know I can’t so why do you keep saying it? (S1).  Participant V: I think for me, probably the best method is to just line myself up with an engagement with someone […] And so that way I go to control myself to being normal […] But if I was left to my own devices, then I don’t know. […] And I think like a normal person would just not go… Because for the average person… exercise wouldn’t be on their mind. (S8).  Participant Z: I have done so much therapy on food that I have come to a point where I stop talking about food being a part of it… I think food is not the issue […] It does not feel normal, normal people do not write down food […] It has been such a long time trying to normalise me (S2).  Participant Z: I feel comfortable eating dinner on my own, there is something about it that I like […] I like to be on my own… I don’t like people to be around. (S2).  Participant Z: So, I’m trying hard to be normal. (S3).  Therapist: Are you sort of thinking that you have rules but there is some flexibility in that for you? Participant Y: … Like yeah, ‘cause I feel like it is kind of like if you don’t do it, you’re going to go crazy or something. (S9).  Participant T: I don’t know… I like being the thin friend. Therapist: You want to be thinner than everyone? Participant T: Yeah. (S1).  Participant T: I know that overall it’s probably a good day… Good in terms of like regular people. (S3).  Participant Q: I get really anxious if I see other people exercising. Therapist: Okay, is that sort of a comparison? Participant Q: Yes. (S1).  EXTRACTS 5 (mid sessions):  Participant X: It does affect your relationships and it puts a strain on things […] [the ED] makes you have more arguments with people more often because they don’t understand you… only you understand yourself and it’s just easier to be alone […] I instantly feel bitter towards really thin people, because I wish I was them. (S10).  Participant X: But I still have to cut out a few good groups from my life which I binge sometimes, so I do not know what is normal. (S10).  Participant X: I smell the salmon, I am eating it but still it is controlled and I am less crazy about it. (S20).  Participant T: I don’t want them to see me eating. (S14).  Participant V: Maybe I was trying to adopt more of a normal person’s perspective in arguing with the eating disorder… I think because there were other people around it was probably more important that I looked or appeared to be more normal. (S12).  Participant Y: It’s that thing… thin people are, like being thin is the ultimate goal, so thin people are the most successful, worthwhile people […] Yeah, cause anything that helps you get thinner is good and anything that doesn’t is bad […] If I’m not doing exercise than I’m not even as good as any of these people. I’m like in a different level of society […] If I don’t exercise it makes me feel unsuccessful and unworthy, yeah it makes me feel lazy, like I often call myself lazy all the time. I actually consider myself to be a lazy person. (S9).  Participant W: I feel normal. I feel huge really […] I’m 44kg, that’s a normal person weight, not an anorexic weight. I’m not anorexic anymore […] I’m seeking treatment but I’m not anorexic. (S14).  Participant W: I am going to be short tempered with the kids if they have done anything wrong. (S14).  Participant Z: No everybody is, not large but on the larger side of normal and that scares me too, because I am obviously smaller than normal and that used to freak me out before. (S15).  Participant Z: I don’t like, I can’t deal with fat people at the moment, still. Remember I told you I feel really sick when I see big people eating lots of food. Fortunately, at lunch time she [coworker] does not eat a lot of food but yeah kind of freaks me out still. (S15).  Participant S: My brother is the middle child, he’s the trouble-maker and the immature one, the lazy one who gets into trouble, and I’m the responsible, mature one… (S13).  EXTRACTS 6 (late sessions):  Participant R: I’d try to avoid doing that to, not worry them or burden […] A lot of my fear of sort of stepping out and doing more thing socially is because… there’s the uncertainty of stepping out of my comfort zone being at home… and meeting new people and the chance that the new people won’t like me or will reject me. (S22).  Therapist: Do you feel like you can open up and be vulnerable to other people? … Participant R: Maybe I am not so good at that. (S22).  Participant Z: I am normally stressed about… if my new friends are judging me. (S32).  Participant Z: I am looking at them going ‘god you look terrible’ and I don’t want to do that again. (S32).  Participant W: … they do not understand what it is… I was like oh you don’t get it. Yeah I don’t need the comments though… The lady’s last comment was oh she is improving, what are you eating, you look so lovely, you’re skinny, you’re fit. (S30). |

**Theme 2: Rebuilding Identities**

| **Subtheme** | **Participant Extract (Session Number)** |
| --- | --- |
| Theme 2a: Shifting Relationship with Oneself | EXTRACTS 7 (early sessions):  Participant Y: Change the mentality about caring so much about my weight and thinking that I have to be so thin, just relaxing a bit more with that and I think the eating normality will follow […] The ultimate goal would be if I didn’t have the ED anymore, I think that’s the ultimate goal. And then the kind of greater goal would be to slowly change my mentality towards being thin, so taking away the importance of that and you know, just relaxing into being the way I am a little bit more or not putting so much focus on that in my life, not having it consume so much of my thoughts. (S1).  Participant Y: … a moderate amount of exercise for your health or something, as opposed to trying to tie it to your worth as a person. (S9).  Participant R: Allow my body to recuperate and allow myself to accept the practical and rational reasons for taking it easy sometimes. (S4).  Participant X: There is a difference between a healthy mind and a healthy body. My body wants to be healthy and happy, but where is my healthy and happy mind? (S1).  Participant Q: I think self-esteem is the main thing. (S1).  EXTRACS 8 (mid and late sessions):  Participant Z: I don’t know what recovered is like, I don’t know what it feels like. (S15).  Participant V: I think I have to really think about… that everything I do I guess is carving a way out of the ED, and that’s something that’s hard for me to believe. (S12).  Participant V: Maybe if I got better at this… maybe the stronger I believe this, the more likely it’s going to ‘beat’ the ED itself. (S21).  Participant V: I need to reduce the level of expectations that I set for myself to break out of that cycle. (S21).  Participant Y: I don’t want to put on weight, but they keep telling me that I have to put on weight to get better, but I need to get rid of all these symptoms. (S24). |
| Theme 2b: Building Life and Identities Outside the AN Identity | EXTRACTS 9 (early sessions):  Participant X: … I used to play a lot of music and write things… I want to go back to only keeping myself busy with things that make me happy and not the ED […] I really like it that I can slow down, sit down with my kids, you know this has really started to come back. (S1).  Participant X: I will basically not let the eating disorder kind of rule your day today. (S1).  Participant Y: I’d like to be able to go out with friends and do that, leading a natural life… I want to be more social, go out more. (S1).  Participant V: And actually to want to probably stay better because I’m not doing this to meet some target so I can get out of hospital. It’s like, for the rest of my life. (S1).  Participant R: They [compulsive exercises] are not helping me with any or either of my goals, or any of my goals really. (S4).  EXTRACTS 10 (mid and late sessions):  Participant S:… that does seem very exciting and I can sort of add another chapter in my life. (S13).  Participant W: I just want to start to be able to not think about it and want to go on with my life without giving it focus. I want to start letting go. (S14).  Participant W: [reading journal extract exploring what recovery would mean for them] Calm and happy, nice, patient, kind, logical, healed, transmit health, compassionate, beautifully strong and fit, relationships, healthy, balanced, happy kids, help others, freedom, picnics, going out for dinners, loving self, socially free and liberated. (S14).  Participant Z: Working together for the goals and crossing them and building a future, it feels so nice to be here. (S32).  Participant S: I think I’ve got a lot more independence, I have a little more confidence to be able to eat around other people and I have been able to go to the yoga class […] You have more control over how you manage meals and everything. (S26). |
